# Can Large Language Models Support University Counseling? Evidence from Perceived Counseling Alliance, Disclosure Willingness, and Risk Recognition

**DOI:** 10.3390/bs16081316

**Published:** 2026-08-03

**Authors:** Jianshan Cheng

**Affiliations:** School of Foreign Languages, Wuhan Institute of Technology, Wuhan 430205, China; 06090902@wit.edu.cn

**Keywords:** large language models, university counseling, perceived counseling alliance, self-disclosure, risk recognition, Chinese university students

## Abstract

The rapid expansion of large language models (LLMs) has created new opportunities for university mental health services, particularly in contexts where counseling demand exceeds available professional resources. This study examined the application of an LLM-based intelligent agent in Chinese university counseling settings, focusing on three key outcomes: perceived counseling alliance, disclosure willingness, and risk recognition. Using a randomized between-subjects experimental design, 388 valid responses were collected from university students and assigned to either an LLM-based counseling condition or a control condition. The LLM-based agent significantly improved perceived counseling alliance and disclosure willingness, and perceived counseling alliance partially mediated the relationship between condition and disclosure. Scenario-based analyses further showed that the LLM-based agent improved general risk recognition and slightly enhanced sensitivity to high-risk cues, although performance remained more limited for crisis-level disclosures. These findings suggest that LLM-based agents are most effective as front-end support tools that facilitate engagement and emotional expression rather than as autonomous crisis detectors. In the context of Chinese university counseling, the study supports a complementary human–AI model in which intelligent agents lower barriers to help-seeking while trained counselors retain responsibility for risk assessment and intervention. Overall, the results contribute to digital mental health research by clarifying both the relational benefits and safety boundaries of LLM-supported counseling.

## 1. Introduction

University students represent a population that is particularly vulnerable to psychological distress due to academic pressure, career uncertainty, interpersonal challenges, and developmental transitions. In recent years, concerns regarding college students’ mental health have intensified worldwide, and China is no exception. National surveys have consistently reported increasing levels of depression, anxiety, academic burnout, and emotional distress among Chinese university students (Y. Liu et al., 2021; Qing et al., 2023). In response, Chinese educational authorities have elevated student mental health to a strategic priority, emphasizing the development of comprehensive psychological support systems within higher education institutions (Ministry of Education of China, 2023).

Despite substantial policy investment, significant challenges remain in university counseling services. Existing studies suggest that although most Chinese universities have established counseling centers, the utilization rate of professional psychological services remains relatively low (Ning et al., 2022). Many students continue to avoid formal counseling because of stigma concerns, fear of negative evaluation, privacy worries, or uncertainty regarding the effectiveness of psychological intervention (W. Chen & Dang, 2026). Furthermore, the growing demand for mental health support has placed considerable pressure on counseling resources, resulting in shortages of qualified counselors and limited service accessibility, particularly during periods of heightened psychological stress (L. Wang & Zhang, 2023). Consequently, there is an urgent need to explore innovative approaches that can complement existing counseling services and reduce barriers to help-seeking.

Recent advances in artificial intelligence (AI), particularly large language models (LLMs), have generated new possibilities for digital mental health support. Since the public release of ChatGPT in late 2022, the adoption of large language models has expanded at an unprecedented pace. ChatGPT became one of the fastest-growing consumer applications in history, reaching more than 100 million monthly active users within two months of launch, and OpenAI reported approximately 800 million weekly active users by 2025. This rapid growth reflects increasing public acceptance of LLM-based conversational systems across education, healthcare, and psychological support settings (OpenAI, 2025). Unlike traditional rule-based chatbots, LLM-based systems can engage in contextually coherent and human-like conversations, making them potentially valuable tools for psychological support and counseling-related applications (Ji et al., 2023). As a result, researchers have increasingly explored the role of LLMs in mental health screening, psychoeducation, emotional support, and counseling assistance (Hua et al., 2025; Balan & Gumpel, 2025).

Importantly, current scholarship does not suggest that AI systems should replace professional counselors. Rather, LLMs are increasingly viewed as supplementary tools that can expand service accessibility, provide immediate support, and facilitate early engagement with mental health resources (Omar et al., 2024; Hua et al., 2025). This perspective is particularly relevant in university settings, where students often seek help during moments of emotional vulnerability but may hesitate to approach human counselors directly. The perceived anonymity, immediacy, and nonjudgmental nature of AI-mediated interaction may lower psychological barriers and encourage initial help-seeking behaviors (Meng & Dai, 2021).

To understand the value of LLM-based counseling systems, it is necessary to move beyond the simple question of whether AI-generated responses appear helpful. Instead, researchers must examine the psychological processes through which such systems influence users. Counseling effectiveness is fundamentally relational. In both traditional and digital counseling contexts, users’ perceptions of being understood, supported, and collaboratively engaged often shape the quality of the counseling experience (Bordin, 1979). Therefore, one key construct for evaluating AI-assisted counseling is perceived counseling alliance, which refers to the collaborative and affective relationship established during the helping process. Emerging evidence suggests that users may develop meaningful working alliances with conversational agents, and such alliances may positively influence engagement and intervention outcomes (Malouin-Lachance et al., 2025).

Another critical process variable is disclosure willingness. Self-disclosure is widely regarded as a prerequisite for effective counseling because counselors can only provide meaningful assistance when individuals are willing to share their emotions, experiences, and concerns. Previous studies have shown that chatbot interactions may facilitate disclosure by reducing social evaluation anxiety and increasing perceived anonymity (Meng & Dai, 2021; Merwin et al., 2025). For university students who may be reluctant to discuss sensitive issues with human professionals, LLM-based agents could function as low-threshold entry points for emotional expression and help-seeking.

However, the potential benefits of AI-supported counseling must be evaluated alongside important safety concerns. Among these concerns, risk recognition is particularly critical. Effective counseling requires the ability to identify psychological crises such as suicidal ideation, self-harm intentions, severe depression, and other high-risk conditions. Although LLMs demonstrate impressive conversational capabilities, recent research indicates that they remain inconsistent in recognizing and responding to crisis-related disclosures (P. Qi et al., 2023; Santos et al., 2026). In some situations, AI systems may provide empathic responses while failing to accurately assess the severity of psychological risk or recommend appropriate intervention strategies, particularly when users disclose suicidal ideation, self-harm intentions, or other crisis-level concerns (Santos et al., 2026; Stamatis et al., 2026). Such limitations raise important questions regarding the appropriate boundaries of AI involvement in mental health care.

Although previous studies have separately examined therapeutic alliance, self-disclosure, and risk detection in AI-mediated environments, relatively little research has investigated these processes within a unified framework, particularly in the context of Chinese university counseling services. Given the increasing demand for mental health support among Chinese college students and the rapid emergence of LLM-based conversational agents, a more comprehensive understanding of these mechanisms is needed.

Therefore, the present study examines the application of an LLM-based intelligent agent in university counseling through three key outcomes: perceived counseling alliance, disclosure willingness, and risk recognition. By integrating these constructs into a single conceptual framework, this study seeks to clarify both the opportunities and limitations of AI-assisted counseling in higher education settings. Specifically, it aims to identify whether LLM-based agents can enhance relational engagement and disclosure while simultaneously examining their capacity to recognize psychological risk. The findings may contribute to the development of hybrid human–AI counseling systems that combine the accessibility of intelligent agents with the professional judgment and ethical responsibility of trained mental health practitioners.

## 2. Literature Review and Hypotheses Development

### 2.1. LLM-Based Intelligent Agents in Mental Health Care

Large language models (LLMs) have rapidly expanded the technical possibilities of digital mental health care, especially in contexts where access to human counselors is limited. Recent reviews show that LLM applications in mental health have been explored across screening, intervention, and counseling-support functions, but their clinical deployment remains constrained by concerns about reliability, privacy, interpretability, and ethical oversight (Hua et al., 2025; Guo et al., 2024). At the same time, domain-specific systems such as ChatCounselor, SoulSpeak, and CA+ suggest that when LLMs are trained or prompted with counseling knowledge, they may provide more context-aware and emotionally responsive interactions than generic chatbots, thereby improving user engagement and perceived usefulness (J. M. Liu et al., 2023; X. Wang et al., 2025; Tang et al., 2026).

However, the same literature also emphasizes that mental health support is not simply a language-generation task. The key challenge is not whether an LLM can produce fluent responses, but whether it can sustain psychologically meaningful interaction while remaining safe, stable, and appropriately bounded. A scoping review of LLMs in mental health care concluded that current systems show promise in accessibility and early support, yet they remain insufficient as stand-alone interventions because of evaluation gaps and unresolved ethical risks (Hua et al., 2025). Consistent with this view, a perspective paper on LLMs in mental health applications stresses that human counselors’ empathy, contextual judgment, and nuanced interpretation remain irreplaceable in high-stakes emotional settings (Ji et al., 2023).

### 2.2. Perceived Counseling Alliance in Human-AI Interaction

A central reason why LLM-based intelligent agents are attracting attention in counseling is their potential to support relational processes rather than only deliver information. In psychotherapy and counseling, therapeutic alliance has long been considered a critical predictor of engagement and outcome. In text-based counseling, researchers have begun to use LLMs not only to generate support but also to assess and model the development of therapeutic relationship quality from conversational transcripts. Recent research has increasingly applied large language models to the analysis of therapeutic processes in counseling settings. For instance, A. Li et al. (2024) developed an LLM-based approach for identifying therapeutic alliance in online text-based counseling. Extending this line of work, A. Li et al. (2026) introduced CARE, an explainable framework that predicts clients’ perceptions of the therapeutic alliance from counseling transcripts while providing interpretable rationales for its predictions. Similarly, the COMPASS framework demonstrated that alliance-building strategies and their development across psychotherapy sessions can be modeled computationally through natural language interactions, highlighting the potential of language-based approaches for capturing relational dynamics in counseling (Lin et al., 2025). These findings indicate that alliance is not an exclusively human-to-human phenomenon in its observable conversational features; rather, it can emerge, at least partially, through the interactional structure of text-mediated exchange.

Building on this literature, LLM-based intelligent agents may foster a preliminary sense of perceived counseling alliance in university settings because they are available, nonjudgmental, and able to maintain conversational continuity. In digital mental health contexts, continuity and responsiveness are especially important because users often seek support in moments of uncertainty and emotional vulnerability. Empirical work on LLM-supported counseling also suggests that when systems are designed with counseling-specific knowledge and interaction strategies, they can improve engagement and perceived professionalism compared with generic conversational models (J. M. Liu et al., 2023; Tang et al., 2026). Accordingly, it is reasonable to expect that a well-designed LLM agent will increase users’ perceptions of collaboration, understanding, and working relationship quality.

**Hypothesis** **1 (H1).** *The use of an LLM-based intelligent agent in college counseling will be positively associated with perceived counseling alliance.*

### 2.3. Disclosure Willingness and Relational Reciprocity

Disclosure willingness is another critical mechanism in counseling. In psychological help-seeking, the extent to which individuals disclose private experiences often determines whether counseling can move beyond surface-level conversation. In computer-mediated settings, disclosure is shaped by perceived anonymity, judgment risk, and the interpersonal style of the interaction. Empirical evidence shows that chatbot self-disclosure can shape users’ own disclosure behavior: when a chatbot displays emotional self-disclosure, users tend to reciprocate with higher-level disclosure, report greater interactional enjoyment, and evaluate the bot more positively as an interaction partner (Meng & Dai, 2021). Similarly, when disclosure is framed as less temporally fixed and less socially exposing, users may feel more willing to share sensitive information with a chatbot (Merwin et al., 2025). These findings are especially relevant in counseling contexts, where the reduction in social threat is often a prerequisite for honest communication.

For university students, the appeal of an LLM-based intelligent agent may lie precisely in its capacity to lower the social cost of disclosure. Students often hesitate to reveal distress because of stigma, self-presentation concerns, or fear of evaluation. A conversational agent that is available on demand, avoids visible emotional reactions, and responds in a nonpunitive tone may create a comparatively safe entry point for initial disclosure. This does not mean that disclosure to a machine is equivalent to disclosure to a human therapist; rather, it suggests that LLMs may function as a transitional medium that helps users articulate concerns before they seek face-to-face support. The counseling literature increasingly recognizes this intermediate role as a realistic design target for digital mental health tools (Hua et al., 2025; Nie et al., 2025).

**Hypothesis** **2 (H2).** *The use of an LLM-based intelligent agent in college counseling will be positively associated with users’ disclosure willingness.*

**Hypothesis** **3 (H3).** *Perceived counseling alliance will be positively associated with users’ disclosure willingness.*

This third hypothesis follows the broader logic of counseling process research: when users experience a stronger sense of collaboration, understanding, and emotional attunement, they are more likely to reveal sensitive personal material. In digital counseling contexts, alliance and disclosure may therefore reinforce each other, forming a relational loop in which early alliance fosters deeper disclosure and deeper disclosure further strengthens perceived alliance. Evidence from text-based counseling and chatbot interaction studies supports this reciprocal interpretation, even though the exact magnitude of the effect may vary by user characteristics and conversation design (Meng & Dai, 2021; A. Li et al., 2024).

### 2.4. Risk Recognition and Safety Boundaries

The third major issue is risk recognition. In counseling, risk detection is not a secondary technical task; it is a core safety requirement. LLMs may be useful in early screening and conversational support, but multiple studies have shown that their performance degrades in high-stakes or ambiguous conditions. A benchmark study on Chinese social media demonstrated that LLMs still lag behind supervised learning models in detecting suicidal risk and cognitive distortions, and the performance gap remained substantial under prompt-only conditions (H. Qi et al., 2025). Another study evaluating the psychological counseling ability of mainstream LLMs found that model performance differs across Chinese and English question sets, underscoring the instability of counseling-related reasoning across language contexts (Peng & Nie, 2025). These findings imply that LLM-based agents cannot yet be treated as reliable stand-alone detectors of psychological crisis.

Evidence regarding the safety of LLMs in mental health contexts remains mixed. Research examining responses to high-risk mental health disclosures has shown that, although advanced models are often capable of generating empathic language, they may fail to consistently recognize risk signals, provide actionable support, or maintain appropriate crisis-oriented engagement. Consequently, current systems have been considered insufficient for meeting clinical expectations in crisis response settings (Santos et al., 2026). Similar concerns emerge from large-scale analyses of real-world interactions. Drawing on more than 20,000 conversations, Stamatis et al. (2026) reported that multilayered safety mechanisms can substantially reduce harmful outputs; however, effective risk mitigation depends not only on model capabilities but also on ongoing monitoring and governance during deployment. Taken together, these studies indicate that risk recognition is where the boundary between assistance and danger becomes most consequential.

Therefore, the present study treats risk recognition as a safety-dependent capability rather than a generic conversational skill. In low- to moderate-risk situations, an LLM-based agent may support screening by eliciting relevant self-report and helping users articulate concern. In contrast, high-risk disclosures such as suicidal ideation, self-harm intent, psychotic symptoms, or severe trauma responses require human escalation and cannot be delegated to the model alone. This logic is consistent with the broader mental health LLM literature, which repeatedly emphasizes that current systems should complement, not replace, professional judgment (Ji et al., 2023; Hua et al., 2025).

**Hypothesis** **4 (H4).** *The use of an LLM-based intelligent agent will improve the recognition of general psychological distress, but its performance will be significantly weaker for high-risk crisis cues than for explicit non-crisis concerns.*

**Hypothesis** **5 (H5).** *Disclosure willingness will positively predict risk recognition accuracy, because richer and more open disclosure provides more diagnostic information for identifying psychological risk.*

This final hypothesis reflects a key process logic in counseling: risk recognition depends not only on the detector’s capability, but also on the quality and completeness of the information disclosed during the conversation. If an LLM-based agent can foster stronger disclosure, it may indirectly improve the visibility of distress signals. Yet this indirect benefit does not eliminate the need for human oversight, especially when the content involves imminent self-harm or other emergency-level concerns. In that sense, the model’s value lies in facilitating safer detection pathways, not in replacing crisis professionals.

### 2.5. Summary of the Conceptual Logic

**Figure 1:**
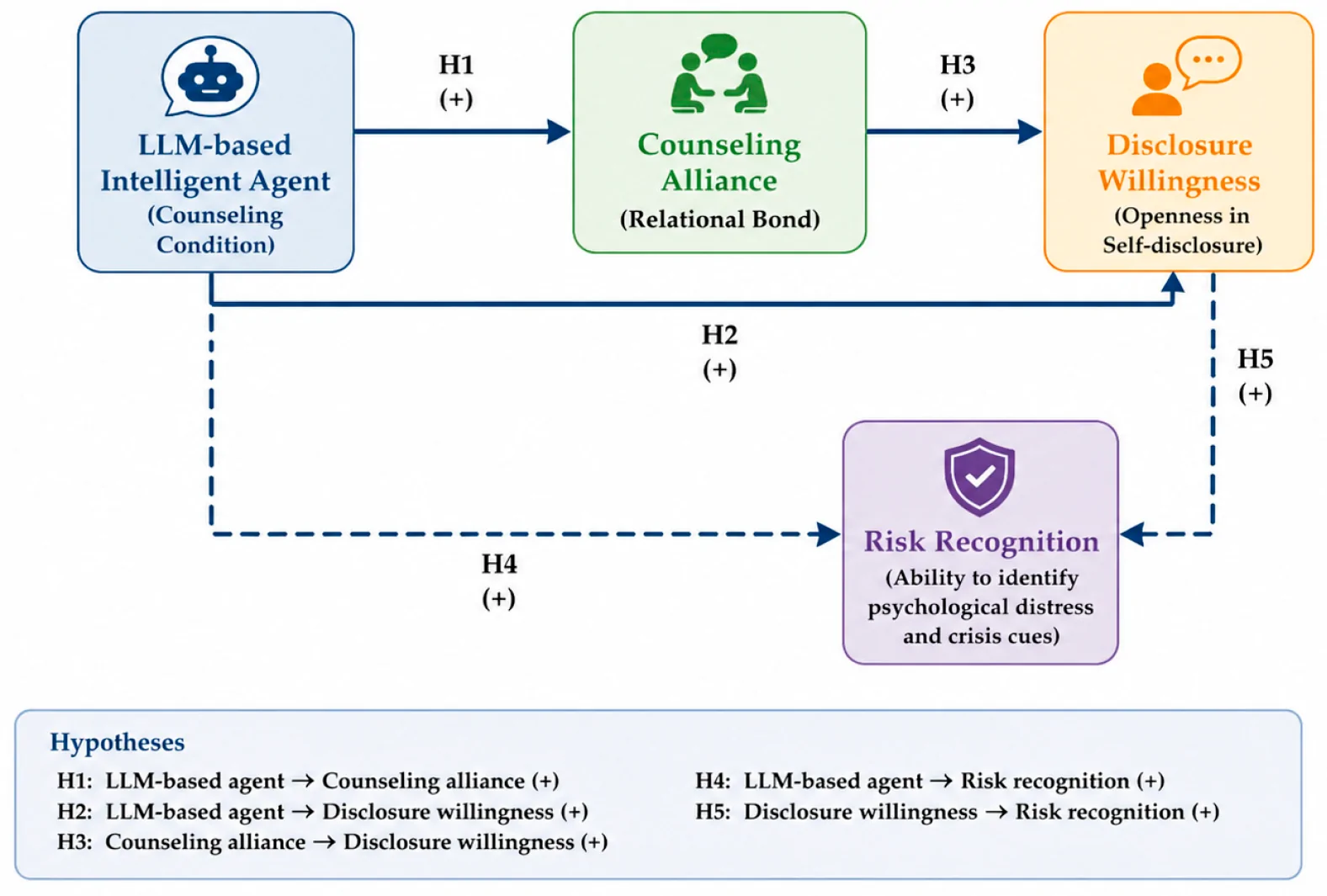
Proposed conceptual framework. Solid arrows indicate direct effects and dashed arrows indicate direct paths to the moderator outcome (rish recognition).

Overall, the literature suggests that LLM-based intelligent agents may be particularly valuable in college counseling when they are designed as relational and supportive tools rather than autonomous therapists. They may enhance perceived counseling alliance by providing a responsive and nonjudgmental interaction space, increase disclosure willingness by lowering social evaluation concerns, and support early risk recognition by eliciting richer self-report. At the same time, the literature is equally clear that crisis-level safety remains a major boundary: current models are still inconsistent in recognizing and responding to high-risk mental health disclosures, making human supervision indispensable (Hua et al., 2025; Santos et al., 2026; Stamatis et al., 2026). To provide a clearer overview of the proposed theoretical relationships, Figure 1 presents the conceptual framework guiding this study and summarizes the hypothesized associations among the LLM-based intelligent agent, perceived counseling alliance, disclosure willingness, and risk recognition.

## 3. Methods

### 3.1. Research Design

This study employed a randomized between-subjects experimental design to examine the psychological effects of a large language model (LLM)-based intelligent agent in university counseling contexts. The design compared an LLM-based counseling condition with a control condition that provided standardized mental health information without interactive dialogue. The primary outcomes were perceived counseling alliance, disclosure willingness, and risk recognition performance. This design was chosen because it allowed the study to capture both relational and safety-related processes that are central to counseling interactions supported by AI-based systems.

### 3.2. Participants and Recruitment

Participants were university students recruited from three higher education institutions in China. Eligibility criteria included being at least 18 years old, being currently enrolled as an undergraduate student, and being able to complete the online counseling simulation independently. Students who were currently receiving inpatient psychiatric treatment or who reported an acute psychological crisis during screening were excluded from the study and were immediately redirected to professional support resources.

A total of 400 questionnaires were distributed online, and 388 valid responses were retained after data screening and exclusion of incomplete or invalid cases. The final sample was balanced across the two experimental conditions, with 196 participants assigned to the LLM-based agent condition and 192 to the control condition. Participation was voluntary, and informed consent was obtained from all participants before the study began.

### 3.3. Procedure

**Figure 2:**
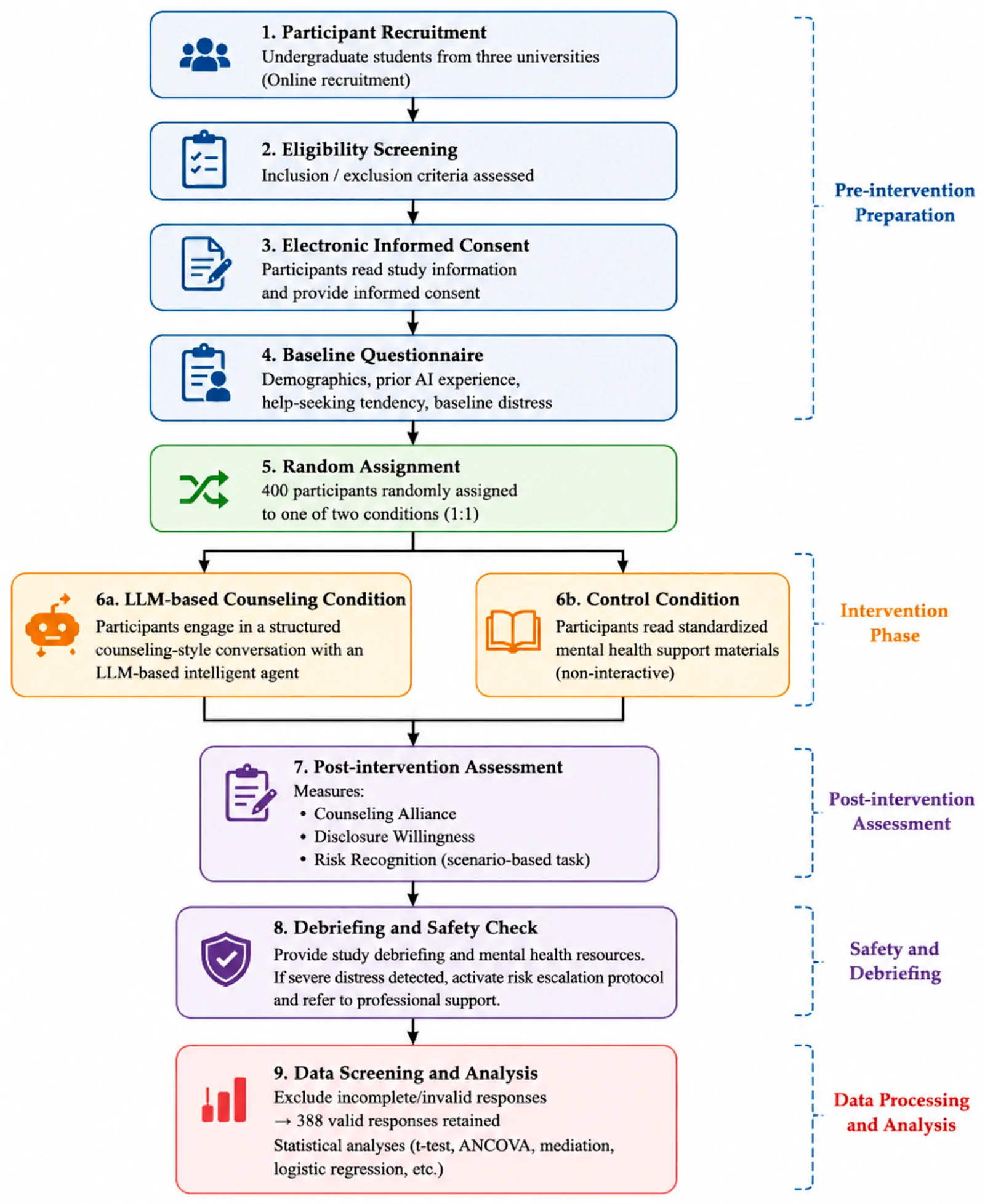
Flowchart of the experimental procedure.

The study employed a randomized experimental design to examine the effects of an LLM-based conversational agent on perceived counseling alliance, disclosure willingness, and psychological risk recognition (Figure 2). After providing informed consent, participants were randomly assigned to either the LLM-based agent condition or the control condition. Participants were informed that the study aimed to examine experiences with different forms of psychological support interaction and that their responses would be used only for research purposes.

In the LLM-based agent condition, participants interacted with a GPT-based large language model configured for supportive counseling-oriented communication. The purpose of the agent was not to provide clinical diagnosis or psychotherapy, but to simulate an initial supportive psychological conversation. The system was designed to facilitate emotional expression and evaluate participants’ perceptions of AI-mediated support.

To improve reproducibility and maintain consistency across participants, a standardized system prompt was developed before data collection. The prompt instructed the LLM to adopt a supportive, respectful, and nonjudgmental communication style. Specifically, the agent was guided to (1) acknowledge and validate users’ emotional experiences, (2) encourage elaboration through open-ended questions, (3) use reflective responses to demonstrate understanding, (4) provide general coping-oriented suggestions when appropriate, and (5) avoid diagnostic judgments, clinical labels, or definitive interpretations of psychological conditions. The same model configuration and prompt framework were applied to all participants assigned to the LLM condition.

The interaction followed a structured but flexible conversation protocol. First, the agent provided an introductory message inviting participants to discuss recent emotional experiences, academic pressures, interpersonal concerns, or other sources of stress. Second, the agent responded to participants’ disclosures through reflective listening, clarification questions, and supportive feedback. Third, the agent encouraged participants to further consider their feelings or coping approaches. Although the overall conversational structure was standardized, the specific dialogue content varied according to participants’ individual responses.

Each interaction lasted approximately 10–15 min. Participants were encouraged to engage naturally with the agent rather than follow a fixed script. After completing the interaction, participants immediately completed questionnaires assessing perceived counseling alliance, disclosure willingness, and related psychological outcomes.

Participants in the control condition completed a comparable psychological support task without interaction with the LLM-based agent. They were presented with the same general instructions regarding emotional reflection and psychological support but did not receive AI-generated conversational responses. This design allowed the study to isolate the effects of AI-mediated interaction rather than the general influence of completing a reflective task.

Following the interaction phase, all participants completed the same outcome measures. In addition, participants completed a scenario-based risk recognition task in which they evaluated hypothetical psychological distress cases. This task was designed to assess the extent to which participants could recognize general psychological risk indicators and high-risk warning signals after exposure to different support conditions.

Overall, the experimental procedure was designed to balance ecological realism and experimental control. While the interaction simulated an initial counseling encounter, it should be noted that it represented a brief, structured AI-mediated conversation rather than a substitute for ongoing professional counseling relationships.

### 3.4. Measures

#### 3.4.1. Perceived Counseling Alliance

Perceived counseling alliance was assessed using an adapted version of the Working Alliance Inventory framework (Bordin, 1979; Horvath & Greenberg, 1989). The original WAI conceptualizes alliance in terms of agreement on goals, agreement on tasks, and affective bond. In the present study, the wording of the items was revised to fit a human–AI counseling context. The adapted items assessed whether participants felt that the agent understood their concerns, whether the interaction felt collaborative, and whether the conversation created a sense of shared purpose and emotional support. Higher scores indicated stronger perceived counseling alliance.

#### 3.4.2. Disclosure Willingness

Disclosure willingness was measured using a self-report scale developed for this study and refined for the digital counseling context. Because no established instrument fully matched the present AI-mediated counseling setting, the item pool was constructed following standard scale-development procedures (DeVellis & Thorpe, 2021) and was written to capture participants’ willingness to share personal emotions, private experiences, and sensitive interpersonal or academic concerns during the interaction. The final disclosure willingness scale consisted of five items, such as “I would be willing to share my personal concerns and emotional difficulties with the agent.” The resulting measure focused on the extent to which participants felt comfortable revealing distress and personal information to the conversational agent. Higher scores reflected greater willingness to disclose.

#### 3.4.3. Risk Recognition Performance

Risk recognition was evaluated through a scenario-based task developed for the present study. The task included standardized counseling vignettes representing three levels of risk: low-risk distress, moderate psychological distress, and high-risk crisis cues. The vignettes were designed to reflect common counseling situations in university settings and to test whether participants could distinguish between ordinary stress, elevated emotional difficulty, and crisis-level indicators. After each vignette, participants were asked to determine whether psychological risk was present, classify its severity, and indicate whether referral or escalation was necessary.

Risk recognition performance was scored using three indices: overall accuracy, sensitivity to high-risk cues, and false negative rate. This approach allowed the study to assess not only whether participants could detect general distress but also whether they could identify the more clinically consequential cases that require human intervention.

#### 3.4.4. Questionnaire Development and Validation

The questionnaire consisted of both adapted and newly developed measures. All self-report items were rated on a five-point Likert scale (1 = strongly disagree, 5 = strongly agree). Perceived counseling alliance was assessed using items adapted from the Working Alliance Inventory (Horvath & Greenberg, 1989). Disclosure willingness items were developed based on previous research on online counseling and chatbot-mediated self-disclosure (Meng & Dai, 2021; Merwin et al., 2025), following the scale-development procedures recommended by DeVellis and Thorpe (2021). Before the formal experiment, the questionnaire was pilot-tested with 35 undergraduate students to evaluate item clarity, readability, and content validity. Based on participants’ feedback and expert review, several wording adjustments were made to improve clarity. The psychometric properties of the final questionnaire were subsequently confirmed through reliability and validity analyses reported in Section 4.1.

### 3.5. Data Analysis

Data analyses were conducted using IBM SPSS Statistics 29.0, IBM AMOS 29.0, and Hayes’ PROCESS macro (Version 4.2). Consistent with standard practice in psychological and behavioral science research, all statistical analyses were conducted using two-tailed tests with a predefined significance level of α = 0.05. This criterion is widely accepted for controlling the probability of Type I error while maintaining adequate statistical sensitivity (Field, 2018). Data screening included checks for missing values, outliers, and normality.

Confirmatory factor analysis (CFA) was performed in AMOS 29.0 to assess the measurement model. Model fit was evaluated using χ^2^/df, CFI, TLI, RMSEA, and SRMR. Construct reliability and validity were examined using Cronbach’s alpha, composite reliability (CR), average variance extracted (AVE), and discriminant validity. Common method bias was assessed using Harman’s single-factor test.

Manipulation checks were conducted using independent-samples *t* tests to examine perceived empathy, supportiveness, and interaction realism across conditions. Baseline equivalence was also tested using *t* tests and chi-square tests.

For hypothesis testing, independent-samples *t* tests and ANCOVA were used to compare conditions on perceived counseling alliance, disclosure willingness, and risk recognition outcomes, controlling for prior AI experience, baseline distress, gender, and academic level. Effect sizes were reported as Cohen’s d and partial eta squared (η^2^*p*).

Risk recognition was further analyzed using logistic regression to predict correct identification of high-risk cases, with odds ratios (ORs) and 95% confidence intervals (CI).

Mediation analysis was conducted using PROCESS Model 4 with 5000 bootstrap samples to test the indirect effect of perceived counseling alliance on the relationship between experimental condition and disclosure willingness.

Finally, robustness and subgroup analyses were performed to examine whether results were consistent across gender, academic level, and prior AI experience.

### 3.6. Ethical Considerations

The study was conducted in accordance with ethical principles for psychological research. Participants were informed that the LLM-based agent was not a substitute for professional counseling and that the task was intended for research purposes only. All data were anonymized and stored securely. To protect participant safety, a pre-established escalation protocol was used for any response indicating severe distress or imminent risk. In such cases, the session was terminated and appropriate referral information was provided immediately. The intelligent agent was also constrained to avoid diagnostic claims, coercive advice, or any response that could compromise participant safety.

## 4. Results

All returned questionnaires were subjected to data quality screening. Responses with substantial missing data, inconsistent answering patterns, or evidence of careless responding were excluded according to the predefined inclusion criteria. Following this screening process, 388 valid questionnaires were retained for the final analyses. The sample included 196 participants in the LLM-based agent condition and 192 participants in the control condition. The mean age of the sample was 21.27 years (SD = 1.75), and 63.1% of participants identified as female. Descriptive analyses indicated that prior AI experience was moderate (M = 3.21, SD = 0.89) and baseline distress was slightly below the scale midpoint (M = 2.89, SD = 0.92).

### 4.1. Measurement Quality and Common Method Bias

Confirmatory factor analysis (CFA) was conducted using AMOS 29.0 to evaluate the adequacy of the measurement model. The hypothesized three-factor measurement model, consisting of perceived counseling alliance, disclosure willingness, and baseline distress, demonstrated an acceptable fit to the data: χ^2^/df = 2.31, CFI = 0.954, TLI = 0.946, RMSEA = 0.058, and SRMR = 0.041. These values met commonly accepted criteria for model adequacy, supporting the validity of the proposed measurement structure.

All standardized factor loadings were significant and ranged from 0.790 to 0.863, indicating satisfactory indicator reliability. As shown in Table 1, all constructs demonstrated strong internal consistency, with Cronbach’s α values ranging from 0.902 to 0.923 and composite reliability (CR) values ranging from 0.903 to 0.923. The average variance extracted (AVE) values ranged from 0.663 to 0.706, exceeding the recommended threshold of 0.50 and supporting convergent validity.

Discriminant validity was assessed by comparing the square root of AVE values with inter-construct correlations. The square root of AVE for each construct exceeded the corresponding correlations with other constructs, indicating satisfactory discriminant validity.

Because all variables were measured using self-report questionnaires, potential common method bias was examined using Harman’s single-factor test. The results showed that the first unrotated factor accounted for 40.50% of the total variance, which was below the conventional 50% criterion. Therefore, common method bias was unlikely to substantially affect the results.

### 4.2. Baseline Equivalence and Sample Descriptives

As shown in Table 2, the sample was predominantly female (245 of 388, 63.1%), with a mean age of 21.27 years (SD = 1.75). Randomization produced comparable groups on age, gender, grade, major, and baseline distress. No between-group differences emerged for age, gender distribution, grade distribution, major distribution, or baseline distress (all *p*s > 0.05). The only baseline imbalance was prior AI experience, which was slightly higher in the LLM-based agent group than in the control group (M = 3.32 vs. 3.09, *t* = 2.51, *p* = 0.012).

### 4.3. Condition Effects on Perceived Counseling Alliance, Disclosure Willingness, and Risk Recognition

Independent-samples *t* tests showed that the LLM-based agent condition outperformed the control condition on all primary outcomes (Table 3). Participants in the LLM-based agent condition reported substantially stronger perceived counseling alliance and greater disclosure willingness, as well as higher general risk accuracy and high-risk sensitivity, and a lower false negative rate. The largest effects were observed for perceived counseling alliance and disclosure willingness, both of which were in the large range.

**Figure 3:**
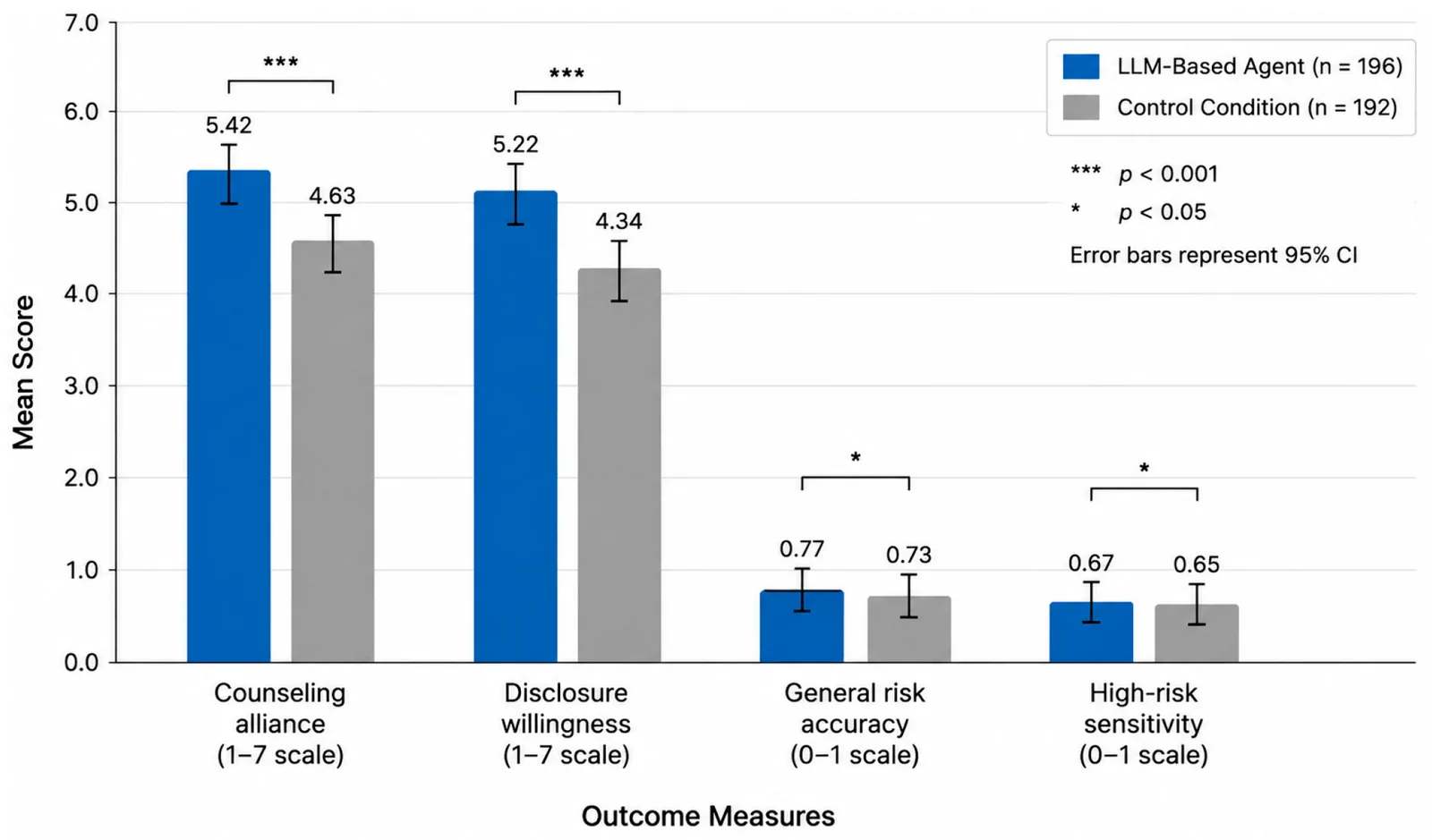
Comparison of Primary Outcomes Between the LLM-Based Agent and Control Conditions. Higher scores indicate better outcomes for counselling alliance and disclosure willingness and higher accuracy/sensitivity for risk recognition outcomes.

Controlling for prior AI experience, baseline distress, gender, and academic level in ANCOVA, the condition effect remained significant for every outcome (Table 4). The LLM-based agent condition continued to predict higher perceived counseling alliance and disclosure willingness, and it also retained a significant advantage on general risk accuracy, high-risk sensitivity, and false negative rate. The magnitude and pattern of these differences across outcome variables are visually summarized in Figure 3.

### 4.4. Correlations and Mediation Analysis

**Figure 4:**
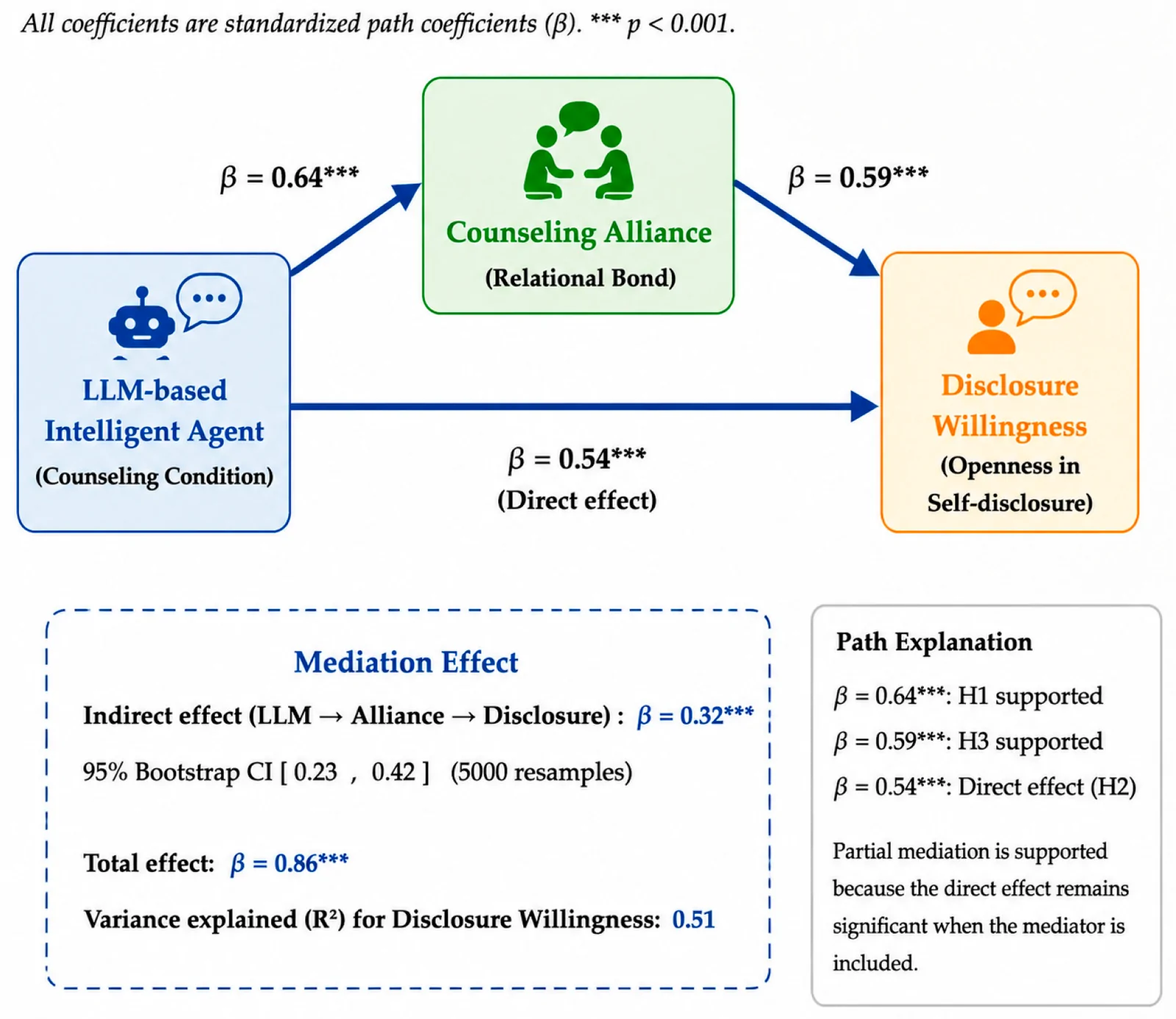
Standardized mediation model. Solid arrows indicate hypothesized direct paths.

As presented in Table 5, Pearson correlations indicated that perceived counseling alliance and disclosure willingness were strongly positively associated (*r* = 0.549, *p* < 0.001). Disclosure willingness was also positively correlated with general risk accuracy (r = 0.176, *p* < 0.001) and high-risk sensitivity (*r* = 0.150, *p* = 0.003), and it was negatively correlated with the false negative rate (*r* = −0.145, *p* = 0.004). perceived counseling alliance showed a smaller but still significant positive association with general risk accuracy (*r* = 0.169, *p* < 0.001). The standardized mediation model and the direct and indirect pathways are presented in Figure 4.

The indirect effect of the LLM-based agent on disclosure willingness through perceived counseling alliance was 0.318, with a 95% bootstrap confidence interval of [0.231, 0.424]. Because the confidence interval did not include zero, the mediation effect was statistically significant. The direct effect remained significant after the mediator was entered, indicating partial mediation.

### 4.5. Robustness and Subgroup Analyses

Robustness analyses controlling for demographic and background variables yielded substantively identical results. Subgroup analyses further demonstrated that the positive effects of the LLM-based agent on perceived counseling alliance and disclosure willingness were significant across gender, academic level, and prior AI experience groups (Table 6). Interaction tests were non-significant (*p*s > 0.05), suggesting that the intervention effects were stable across participant characteristics.

## 5. Discussion

This study examined the application of a large language model (LLM)-based intelligent agent in university counseling contexts, focusing on perceived counseling alliance, disclosure willingness, and risk recognition. Overall, the findings suggest that the LLM-based conversational agent was particularly effective in improving relational engagement and disclosure, while its advantage in high-risk detection remained comparatively limited. In the experimental comparison, participants in the LLM-based condition reported markedly stronger perceived counseling alliance and disclosure willingness than those in the control condition, and the mediation analysis further indicated that alliance was a key pathway linking condition to disclosure. At the same time, although the LLM-based agent improved general risk recognition accuracy and modestly increased sensitivity to high-risk cues, the effect was small, and high-risk sensitivity remained only moderate. This pattern suggests that the agent was better at opening the conversation than at reliably handling the most clinically consequential risk cues.

### 5.1. Why the LLM-Based Agent Enhanced Perceived Counseling Alliance

The strongest and most robust result of the present study was the increase in participants’ perceived counseling alliance following a brief interaction with the LLM-based agent. This finding should not be interpreted as evidence that participants developed a therapeutic relationship equivalent to that established in professional counseling. Rather, it suggests that AI-mediated conversations can generate certain alliance-related perceptions, such as feeling understood, supported, and engaged during an initial interaction. This is consistent with the broader digital mental health literature showing that users can develop meaningful relational bonds with conversational agents when the interaction is responsive, continuous, and perceived as understanding rather than evaluative. Recent work has shown that a digital therapeutic alliance can emerge in mental health chatbots, and that users are capable of perceiving a working relationship with AI systems when the dialogue feels structured, empathic, and goal-oriented (Beatty et al., 2022; Xu et al., 2025). Experimental evidence also suggests that emotionally responsive chatbot behaviors can enhance user satisfaction and reuse intention, which is consistent with the idea that relational cues matter even when the partner is a machine (Park et al., 2023).

One plausible explanation is that the LLM-based agent reduced the interpersonal “cost” of being a help-seeker. In a conventional university counseling appointment, a student may arrive already worrying about how they will be judged, whether the counselor will think their problem is “not serious enough,” or whether the conversation will become too exposed too quickly. By contrast, an LLM agent can offer a tone that is consistently nonjudgmental, slow the pace of disclosure, and return immediate reflective prompts. In that sense, the alliance effect may not mean that students believed the agent was a human counselor; rather, it may indicate that they experienced the interaction as emotionally safe, organized, and responsive. This interpretation is compatible with prior findings that alliance in text-based or AI-mediated counseling is strongly shaped by conversational continuity and perceived responsiveness (Beatty et al., 2022; Xu et al., 2025).

A concrete example helps clarify the mechanism. Imagine a first-year student who has recently entered university, feels homesick, and is reluctant to talk to a real counselor because he worries that “this is too trivial.” In a face-to-face setting, he may minimize his concerns. In an LLM-based dialogue, however, the agent can immediately respond with a neutral prompt such as, “That sounds difficult. Can you tell me what part of this feels hardest for you?” The student is then more likely to feel that the system is listening and that the conversation is moving at his own pace. The alliance score rises not because the agent has human warmth in the full clinical sense, but because it successfully simulates core process features of early counseling contact. That is precisely the kind of “entry-level relational safety” that university counseling systems often struggle to provide at scale.

### 5.2. Why the LLM-Based Agent Increased Disclosure Willingness

The second major finding was the strong increase in disclosure willingness. This result is theoretically important because it suggests that the benefits of the LLM-based agent were not limited to subjective impressions of the interaction; they also translated into a behavioral readiness to reveal personal and emotional information. Previous studies have shown that self-disclosure to digital agents is shaped by perceived anonymity, reduced judgment risk, and the interpersonal style of the chatbot. Chatbots that display emotional disclosure or supportive reciprocity tend to elicit greater user disclosure, and emotionally meaningful disclosure itself is associated with positive relational and psychological outcomes (Ho et al., 2018; Meng & Dai, 2021; Park et al., 2023). Recent work has also suggested that self-disclosure to robots or relational agents can be a meaningful coping route for distress, especially when people are not yet ready to disclose to humans (Laban et al., 2025; Saha et al., 2025).

The mediation result strengthens this interpretation. Perceived counseling alliance partially explained the condition–disclosure link, which means that the agent was not simply lowering barriers in an abstract sense; it was doing so by creating a relational frame in which students felt understood enough to speak more openly. In practical terms, this makes sense. A student is unlikely to disclose more simply because a system is available. What appears to matter is whether the interaction feels collaborative, stable, and nonpunitive. Once that perception is established, disclosure becomes easier because the student no longer needs to spend cognitive effort on impression management. The present data suggest that alliance functioned as the psychological bridge between “having access to an AI agent” and “being willing to say something personal.”

This pattern is particularly evident in China, where many university students are aware of psychological counseling services but still do not actively utilize them. Prior studies have identified several persistent barriers within campus counseling systems, including underdeveloped service infrastructure, uneven levels of professional capacity among counselors, discomfort or resistance during counseling interactions, and limited mental health literacy among students (Feng, 2025; C. H. Li, 2022; J. Chen & Liu, 2022). At the same time, research has suggested that online counseling and other digital support channels may help alleviate these constraints by reducing access barriers and expanding service availability (Y. Liu et al., 2021; C. H. Wang, 2010). Within this context, the present finding is understandable: for students who are hesitant to engage directly in formal counseling settings, LLM-based conversational systems may serve as a low-threshold entry point, providing a space where initial concerns can be articulated before individuals consider transitioning to face-to-face psychological support.

A second example may clarify this point. Consider a sophomore who has been struggling with exam pressure and family conflict but has repeatedly told roommates that “everything is fine.” In a counseling office, she may feel that admitting stress would expose weakness. In an LLM conversation, however, the system can invite disclosure without visible social consequences. Once the student starts describing the situation, the act of naming the stressor may itself reduce emotional burden, which in turn further increases disclosure. This recursive process fits well with the partial mediation pattern observed in the present study and suggests that alliance and disclosure may reinforce each other over the course of the interaction.

### 5.3. Why Risk Recognition Improved Only Modestly

The third major finding was more nuanced. The LLM-based agent improved overall risk recognition and slightly improved sensitivity to high-risk cues, but the effect was modest and high-risk sensitivity remained only moderate. This is an important boundary condition. The data suggest that LLMs are helpful for broad engagement and preliminary screening, but they are not yet reliable enough to serve as stand-alone crisis detectors. The result is consistent with recent work showing that LLMs can still underperform supervised approaches on suicidal-risk and cognitive-distortion detection in Chinese social media, and that counseling-related reasoning can vary substantially across Chinese and English question sets (H. Qi et al., 2025; Peng & Nie, 2025). Recent safety evaluations likewise show that even when models sound empathic, they may still fail to acknowledge risk consistently, offer concrete support, or maintain sufficiently safe crisis responses (Santos et al., 2026). Ecological audits of real conversations further indicate that layered safeguards and continuous monitoring are more important than benchmark performance alone (Stamatis et al., 2026).

The pattern in the current study can be interpreted in light of how psychological risk is typically expressed in university settings. High-risk disclosures are often not explicit. A student may write, “I’m tired,” “I don’t know what the point is,” or “I just want to sleep and not wake up,” without directly naming suicidal intent. Human counselors use context, tone shifts, prior history, and follow-up questioning to infer severity. An LLM can often recognize surface distress, but it may miss the difference between ordinary exhaustion and crisis-level hopelessness, especially when the language is ambiguous or culturally indirect. That is why the agent’s performance improved more clearly on general distress than on crisis cues. In other words, the system seems better at hearing “something is wrong” than at deciding “this requires immediate escalation.”

This is precisely where the boundary between assistance and danger becomes most consequential. The present findings suggest a layered workflow rather than a replacement model. For example, in a university counseling service, an LLM agent could be used as a front-end screener: it might gather initial descriptions, ask clarifying questions, and flag responses that warrant human review. But once content suggests self-harm intent, psychotic symptoms, severe dissociation, or imminent danger, the system must stop short of autonomous judgment and hand the case over to a trained counselor. This hybrid logic is already consistent with the broader Chinese discussion on digital counseling and online mental health support, which emphasizes both the usefulness of new channels and the continuing need for professional authority and crisis protocols (Y. Liu et al., 2021; Feng, 2025).

Importantly, the present findings should be interpreted within the boundaries of the experimental setting. The risk recognition task was based on standardized scenarios rather than spontaneous disclosures occurring in real counseling relationships. In actual counseling contexts, psychological risk assessment requires integration of longitudinal information, behavioral observations, contextual understanding, and professional clinical judgment. Therefore, the current findings should be interpreted as evidence regarding the potential supportive role of LLM-based systems in structured assessment situations rather than direct evidence of clinical effectiveness.

### 5.4. Theoretical Implications

The present findings contribute to the literature in several ways. First, they extend the concept of perceived counseling alliance to human–AI counseling contexts. Traditionally, alliance has been viewed as a collaborative relationship established between counselor and client (Bordin, 1979). The current results suggest that users can experience certain relational perceptions with an LLM-based agent, including perceived collaboration, responsiveness, and understanding during short-term interactions. This finding supports the view that alliance is shaped not only by the human characteristics of the helper but also by interactional qualities such as responsiveness, continuity, and perceived support.

Second, the mediation results clarify the mechanism through which AI-assisted counseling promotes disclosure willingness. perceived counseling alliance partially mediated the relationship between the experimental condition and disclosure, indicating that users are more willing to share personal concerns when they perceive the interaction as supportive and collaborative. This finding enriches existing counseling process theories by suggesting that AI systems may facilitate disclosure through relational pathways similar to those observed in traditional counseling settings.

Third, the relatively modest improvement in high-risk recognition highlights an important boundary condition of LLM applications in mental health care. While the agent effectively enhanced engagement and disclosure, its performance in detecting crisis-level cues remained limited. This finding suggests that LLMs are better suited to supporting early-stage interaction and emotional expression than to making complex clinical judgments. Theoretically, it reinforces the distinction between conversational competence and professional risk assessment, emphasizing that the latter still relies heavily on human expertise.

Finally, the findings support a complementary rather than substitutional view of AI in counseling. Rather than replacing human counselors, LLM-based agents appear most valuable as front-end support tools that facilitate help-seeking, emotional articulation, and preliminary screening. This perspective contributes to emerging theories of hybrid human–AI mental health systems, in which AI enhances accessibility and engagement while trained professionals retain responsibility for diagnosis, crisis management, and intervention.

### 5.5. Practical Implications

The present findings provide several practical implications for the application of LLM-based counseling agents in university mental health services. First, the significant improvements in perceived counseling alliance and disclosure willingness suggest that LLM-based agents may serve as effective entry points for students who are reluctant to seek traditional counseling. In many Chinese universities, concerns about stigma, privacy, and negative evaluation continue to discourage help-seeking. An AI-based agent can reduce these barriers by offering immediate, anonymous, and nonjudgmental interaction, thereby encouraging students to discuss emotional concerns at an earlier stage.

Second, the mediation analysis showed that perceived counseling alliance partially explained the relationship between the intervention and disclosure willingness. This finding highlights the importance of relational design in AI-supported counseling. Rather than functioning solely as information providers, conversational agents should be designed to establish rapport through empathic responses, reflective listening, and supportive follow-up questions. Building a sense of understanding and collaboration may be essential for encouraging students to share personal experiences and psychological difficulties.

Third, the results of the scenario-based task indicate that the LLM-based agent performed better in identifying general psychological distress than in recognizing high-risk crisis cues. Although overall risk recognition improved, the relatively modest gain in high-risk sensitivity suggests that current systems should not be used as autonomous crisis-assessment tools. Instead, universities should adopt a human-in-the-loop model in which AI agents assist with preliminary screening and information collection, while trained counselors remain responsible for risk evaluation and intervention decisions.

Fourth, universities should establish systematic governance mechanisms before implementing AI-supported counseling services. These mechanisms should include clear data privacy policies, transparent communication regarding AI limitations, regular monitoring of model performance, and predefined escalation procedures for high-risk cases. Such governance structures can help ensure that technological convenience does not compromise ethical responsibility or student safety.

Fifth, AI counseling systems should be developed with attention to individual differences among students. Students vary in AI familiarity, psychological literacy, help-seeking attitudes, and willingness to interact with non-human agents. Therefore, universities may consider providing different levels of AI-assisted support, ranging from general emotional support and psychoeducation to guided referral services. A personalized implementation strategy may improve acceptance and maximize the complementary value of AI within campus mental health systems.

Overall, LLM-based counseling agents should be understood as supportive infrastructures rather than replacements for professional psychological services. Their greatest value lies in expanding accessibility, facilitating early emotional expression, and improving pathways toward appropriate human intervention.

### 5.6. Limitations and Future Directions

Several limitations should be acknowledged. First, the experimental nature of the study limits its ecological validity. Although the randomized design allowed causal inference regarding immediate psychological responses to an LLM-based interaction, the simulated counseling environment differs from authentic counseling practice. Real counseling involves repeated sessions, evolving therapeutic relationships, complex emotional dynamics, and professional decision-making processes that cannot be fully captured within a brief experimental interaction. Therefore, future research should examine LLM-supported counseling through longitudinal designs, field experiments, and evaluations conducted within actual university counseling services.

Second, the study focused on immediate post-interaction outcomes. While the results indicate that the LLM-based agent enhanced perceived counseling alliance and disclosure willingness in the short term, it remains unclear whether these effects translate into sustained help-seeking behavior or improved mental health outcomes. Longitudinal studies are needed to examine the durability of these effects over time.

Third, risk recognition was assessed using structured vignettes. Although this approach ensured comparability across participants, real-world psychological crises are often expressed indirectly and require nuanced contextual interpretation. Consequently, the current findings may overestimate the effectiveness of risk detection in actual counseling situations. Future studies should employ more ecologically valid materials, such as real conversation transcripts or field-based evaluations, to assess the safety and reliability of LLM-assisted counseling systems.

Another limitation concerns the cultural and contextual specificity of the sample. Participants in this study were exclusively Chinese university students, whose perceptions of psychological counseling and AI-based support may be shaped by particular social and cultural conditions. For example, concerns regarding stigma, privacy, and social evaluation may influence students’ willingness to disclose psychological difficulties, while increasing familiarity with digital technologies may affect acceptance of AI-mediated interactions.

Therefore, the present findings should be interpreted within the context of Chinese higher education rather than assumed to represent universal patterns. Future research should examine whether similar effects emerge among students from different cultural backgrounds, educational systems, and levels of AI familiarity. Cross-cultural studies may further clarify how cultural norms regarding help-seeking, trust in technology, and perceptions of human versus AI support influence responses to AI-assisted psychological services.

## 6. Conclusions

This study investigated the role of an LLM-based intelligent agent in university counseling by examining its effects on perceived counseling alliance, disclosure willingness, and risk recognition. The findings demonstrate that LLM-supported counseling significantly enhances perceived counseling alliance and students’ willingness to disclose personal concerns. Moreover, perceived counseling alliance serves as an important psychological mechanism explaining why AI-based interactions promote greater openness.

At the same time, the findings reveal important limitations regarding safety-critical applications. Although the LLM-based agent improved general risk recognition, its ability to identify high-risk crisis cues remained relatively limited. Therefore, current AI counseling systems should not be considered substitutes for professional counselors but rather complementary tools that facilitate initial engagement, emotional expression, and preliminary screening.

The study contributes to emerging research on human–AI mental health interaction by showing that relational qualities such as perceived understanding and collaboration can extend beyond traditional human counseling contexts. For university practice, the findings support a hybrid counseling model in which AI technologies improve accessibility while trained professionals maintain responsibility for ethical judgment and crisis intervention. Future research should further examine long-term outcomes and real-world implementation of AI-assisted counseling systems across diverse populations and institutional settings.

## Figures and Tables

**Table 1:** Measurement Model Fit, Reliability, and Validity Results.

Construct	Items	Factor Loading	Cronbach’s α	CR	AVE	χ^2^/df	CFI	TLI	RMSEA/SRMR
Measurement model	-	-	-	-	-	2.31	0.954	0.946	0.058/0.041
Perceived counseling alliance	5	0.790–0.835	0.907	0.908	0.663	-	-	-	-
Disclosure willingness	5	0.830–0.855	0.923	0.923	0.706	-	-	-	-
Baseline distress	4	0.816–0.863	0.902	0.903	0.699	-	-	-	-

Note. CR = composite reliability; AVE = average variance extracted.

**Table 2:** Baseline Demographic and Psychological Variable Comparisons Between Experimental and Control Groups.

Variable	LLM-Based Agent M (SD)	Control M (SD)	*t*	*p*
Age	21.31 (1.74)	21.22 (1.78)	0.49	=0.623
Prior Ai Experience	3.32 (0.96)	3.09 (0.82)	2.51	=0.012
Baseline Distress	2.94 (0.92)	2.84 (0.92)	1.11	=0.269

**Table 3:** Independent Samples *t*-Test Comparisons of Primary Outcome Variables by Experimental Condition.

Outcome	LLM-Based Agent M (SD)	Control M (SD)	*t*	*p*	Cohen’s d
perceived counseling alliance	5.42 (0.60)	4.63 (0.59)	13.22	<0.001	1.34
Disclosure willingness	5.22 (0.70)	4.34 (0.72)	12.21	<0.001	1.24
General risk accuracy	0.77 (0.07)	0.73 (0.08)	6.13	<0.001	0.62
High-risk sensitivity	0.67 (0.10)	0.65 (0.11)	2.29	=0.022	0.23
False negative rate	0.33 (0.10)	0.35 (0.11)	−2.08	=0.038	−0.21

**Table 4:** ANCOVA Results Predicting perceived counseling alliance, Disclosure Willingness, and Risk Recognition Outcomes.

Outcome	B	SE	F	*p*	Partial η^2^
perceived counseling alliance	0.780	0.060	166.28	<0.001	0.305
Disclosure willingness	0.859	0.072	143.63	<0.001	0.275
General risk accuracy	0.046	0.007	37.39	<0.001	0.090
High-risk sensitivity	0.027	0.010	6.66	=0.010	0.017
False negative rate	−0.026	0.011	5.86	=0.016	0.015

**Table 5:** Mediation Analysis Results of perceived counseling alliance as the Mediator Variable.

Path	B	SE	*p*	Interpretation
Condition -> perceived counseling alliance	0.780	0.060	<0.001	Significant
perceived counseling alliance -> Disclosure	0.406	0.057	<0.001	Significant
Condition -> Disclosure (direct)	0.543	0.081	<0.001	Significant
Indirect effect	0.318	Bootstrapped	[0.231, 0.424]	Partial mediation

**Table 6:** Subgroup Analysis of Intervention Effects Across Gender and Prior AI Experience Levels.

Subgroup	Perceived Counseling Alliance (d)	Disclosure Willingness (d)	*p*
Male	1.28	1.17	<0.001
Female	1.37	1.29	<0.001
Low AI experience	1.29	1.21	<0.001
High AI experience	1.35	1.27	<0.001

## Data Availability

The data that support the findings of this study are available in a public repository. The dataset is hosted on Google Drive and can be accessed at: https://drive.google.com/file/d/1uJ8BbhnYyEZWEPXVR7MoJQbE7ZWlGel6/view?usp=sharing (accessed on 13 July 2026); These data are openly available for research purposes without restriction.

## References

[B1-behavsci-16-01316] Balan R., Gumpel T. P. (2025). ChatGPT clinical use in mental health care: Scoping review of empirical evidence. JMIR Mental Health.

[B2-behavsci-16-01316] Beatty C., Malik T., Meheli S., Sinha C. (2022). Evaluating the therapeutic alliance with a free-text CBT conversational agent (Wysa): A mixed-methods study. Frontiers in Digital Health.

[B3-behavsci-16-01316] Bordin E. S. (1979). The generalizability of the psychoanalytic concept of the working alliance. Psychotherapy: Theory, Research & Practice.

[B4-behavsci-16-01316] Chen J., Liu F. E. (2022). Da xue sheng zhuan ye xin li qiu zhu xing wei de zu ai yin su yu cu jin lu jing [Barriers and promotion paths of professional psychological help-seeking behavior among college students]. Chinese Mental Health Journal.

[B5-behavsci-16-01316] Chen W., Dang J. (2026). Mental health malleability beliefs and help-seeking intentions among Chinese college students: The mediating role of help-seeking self-stigma and the moderating role of depression. Frontiers in Psychology.

[B6-behavsci-16-01316] DeVellis R. F., Thorpe C. T. (2021). Scale development: Theory and applications.

[B7-behavsci-16-01316] Feng Z. Y. (2025). “Wu wei yi ti” rong he shi xin li yu ren mo shi de jian gou yu shi jian [Construction and practice of the integrated “five-in-one” psychological education model]. Studies in Ideological Education.

[B8-behavsci-16-01316] Field A. (2018). Discovering statistics using IBM SPSS statistics.

[B9-behavsci-16-01316] Guo Z., Lai A., Thygesen J. H., Farrington J., Keen T. (2024). Large language models for mental health applications: A systematic review. JMIR Mental Health.

[B10-behavsci-16-01316] Ho A., Hancock J., Miner A. S. (2018). Psychological, relational, and emotional effects of self-disclosure after conversing with a chatbot. Journal of Communication.

[B11-behavsci-16-01316] Horvath A. O., Greenberg L. S. (1989). Development and validation of the working alliance inventory. Journal of Counseling Psychology.

[B12-behavsci-16-01316] Hua Y., Na H., Li Z., Liu F., Fang X., Clifton D., Torous J. (2025). A scoping review of large language models for generative tasks in mental health care. npj Digital Medicine.

[B13-behavsci-16-01316] Ji Z., Lee N., Frieske R., Yu T., Su D., Xu Y., Ishii E., Bang Y. J., Madotto A., Fung P. (2023). Survey of hallucination in natural language generation. ACM Computing Surveys.

[B14-behavsci-16-01316] Laban G., Morrison V., Kappas A., Cross E. S. (2025). Coping with emotional distress via self-disclosure to robots: An intervention with caregivers. International Journal of Social Robotics.

[B15-behavsci-16-01316] Li A., Lu Y., Song N., Zhang S., Ma L., Lan Z., Al-Onaizan Y., Bansal M., Chen Y.-N. (2024). Understanding the therapeutic relationship between counselors and clients in online text-based counseling using LLMs. Findings of the association for computational linguistics: EMNLP 2024.

[B16-behavsci-16-01316] Li A., Wang C., Lu Y., Xu R., Ma L., Lan Z. (2026). CARE: An explainable computational framework for assessing client-perceived therapeutic alliance using large language models. arXiv.

[B17-behavsci-16-01316] Li C. H. (2022). Da xue sheng xin li jian kang su yang de ti sheng ce lüe yan jiu [Strategies for improving mental health literacy among university students]. Journal of Heilongjiang Institute of Teacher Development.

[B18-behavsci-16-01316] Lin B., Bouneffouf D., Landa Y., Jespersen R., Corcoran C., Cecchi G. (2025). COMPASS: Computational mapping of patient-therapist alliance strategies with language modeling. Translational Psychiatry.

[B19-behavsci-16-01316] Liu J. M., Li D., Cao H., Ren T., Liao Z., Wu J. (2023). ChatCounselor: A large language model for mental health support. Paper presented at conference contribution.

[B20-behavsci-16-01316] Liu Y., Li B., Hao F., Wang B., Zhu J., Liu D., Zhai J., Chen M., Wang J. (2021). Associations between borderline personality disorder features and the risk of first onset major depressive disorder: Findings from a 2-year longitudinal study in a sample of first-year university students in China. Journal of Affective Disorders.

[B21-behavsci-16-01316] Malouin-Lachance A., Capolupo J., Laplante C., Hudon A. (2025). Does the digital therapeutic alliance exist? Integrative review. JMIR Mental Health.

[B22-behavsci-16-01316] Meng J., Dai Y. N. (2021). Emotional support from AI chatbots: Should a supportive partner self-disclose or not?. Journal of Computer-Mediated Communication.

[B23-behavsci-16-01316] Merwin E. R., Hagen A. C., Keebler J. R., Forbes C. (2025). Self-disclosure to AI: People provide personal information to AI and humans equivalently. Computers in Human Behavior: Artificial Humans.

[B24-behavsci-16-01316] Ministry of Education of China (2023). Special action plan for comprehensively strengthening and improving student mental health in the new era (2023–2025).

[B25-behavsci-16-01316] Nie J., Shao H., Fan Y., Shao Q., You H., Preindl M., Jiang X. (2025). LLM-based conversational AI therapist for daily functioning screening and psychotherapeutic intervention via everyday smart devices. ACM Transactions on Computing for Healthcare.

[B26-behavsci-16-01316] Ning X., Wong J. P.-H., Huang S., Fu Y., Gong X., Zhang L., Hilario C., Fung K. P.-L., Yu M., Poon M. K.-L., Cheng S., Gao J., Jia C.-X. (2022). Chinese university students’ perspectives on help-seeking and mental health counseling. International Journal of Environmental Research and Public Health.

[B27-behavsci-16-01316] Omar M., Soffer S., Charney A. W., Landi I., Nadkarni G. N., Klang E. (2024). Applications of large language models in psychiatry: A systematic review. Frontiers in Psychiatry.

[B28-behavsci-16-01316] OpenAI (2025). The state of enterprise AI.

[B29-behavsci-16-01316] Park G., Chung J., Lee S. (2023). Effect of AI chatbot emotional disclosure on user satisfaction and reuse intention for mental health counseling: A serial mediation model. Current Psychology.

[B30-behavsci-16-01316] Peng F., Nie J. (2025). Psychological counseling ability of large language models. arXiv.

[B31-behavsci-16-01316] Qi H., Fu G., Li J., Song C., Zhai W., Luo D., Liu S., Yu Y., Yang B., Zhao Q. (2025). Supervised learning and large language model benchmarks on mental health datasets: Cognitive distortions and suicidal risks in Chinese social media. Bioengineering.

[B32-behavsci-16-01316] Qi P., Wang H., Chen J. (2023). Benchmarking large language models for suicide risk detection in Chinese social media. Proceedings of the 2023 conference on empirical methods in natural language processing.

[B33-behavsci-16-01316] Qing Y., Li Z., Zhang Y. (2023). Changes in mental health among Chinese university students before and during campus lockdowns due to the COVID-19 pandemic: A three-wave longitudinal study. Frontiers in Psychiatry.

[B34-behavsci-16-01316] Saha K., Yoo D. W., Das Swain V., De Choudhury M. (2025). Mental wellbeing effects of disclosing life events on social media. Scientific Reports.

[B35-behavsci-16-01316] Santos J. M., Shah S., Gupta A., Mann A., Vaz A., Caldwell B. E., Scholz R., Awad P., Allemandi R., Faust D., Banka H., Rousmaniere T. (2026). Evaluating the clinical safety of large language models in response to high-risk mental health disclosures. Practice Innovations.

[B36-behavsci-16-01316] Stamatis C. A., Meyerhoff J., Zhang R., Tieleman O., Malgaroli M., Hull T. D. (2026). Beyond simulations: What 20,000 real conversations reveal about mental health AI safety.

[B37-behavsci-16-01316] Tang Y., Kang Y., Wang Y., Wang T., Zhong C., Gong J. (2026). A counselor-inspired agent framework for AI counselors to enhance client engagement. Technology in Society.

[B38-behavsci-16-01316] Wang C. H. (2010). Gao zhi yuan xiao xin li jian kang jiao yu de you hua yuan ze yu lu jing xuan ze [Optimization principles and path selection of mental health education in vocational colleges]. Education and Vocation.

[B39-behavsci-16-01316] Wang L., Zhang Q. (2023). Xian dai da xue sheng xin li jian kang fu wu de kun jing yu gai jin lu jing [Dilemmas and improvement paths of mental health services for contemporary college students]. Journal of Higher Education Management.

[B40-behavsci-16-01316] Wang X., Zhou Y., Zhou G. (2025). The application and ethical implication of generative AI in mental health: Systematic review. JMIR Mental Health.

[B41-behavsci-16-01316] Xu Z., Lee Y. C., Stasiak K., Warren J., Lottridge D. (2025). The digital therapeutic alliance with mental health chatbots: Diary study and thematic analysis. JMIR Mental Health.

